# Antibodies to SARS-CoV-2 and risk of past or future sick leave

**DOI:** 10.1038/s41598-021-84356-w

**Published:** 2021-03-04

**Authors:** Joakim Dillner, K. Miriam Elfström, Jonas Blomqvist, Carina Eklund, Camilla Lagheden, Sara Nordqvist-Kleppe, Cecilia Hellström, Jennie Olofsson, Eni Andersson, August Jernbom Falk, Sofia Bergström, Emilie Hultin, Elisa Pin, Anna Månberg, Peter Nilsson, My Hedhammar, Sophia Hober, Johan Mattsson, Laila Sara Arroyo Mühr, Kalle Conneryd Lundgren

**Affiliations:** 1 Karolinska University Laboratory, Karolinska University Hospital, 141 86 Stockholm, Sweden; 2 Division of Affinity Proteomics, Department of Protein Science, KTH Royal Institute of Technology, SciLifeLab, 171 65 Stockholm, Sweden; 3 Division of Protein Technology, Department of Protein Science, KTH Royal Institute of Technology, Albanova, 144 21 Stockholm, Sweden; 4 Karolinska University Hospital, 141 86 Stockholm, Sweden

**Keywords:** Infectious diseases, Epidemiology, Epidemiology, Population screening, Infection, SARS-CoV-2

## Abstract

The extent that antibodies to SARS-CoV-2 may protect against future virus-associated disease is unknown. We invited all employees (n = 15,300) at work at the Karolinska University Hospital, Stockholm, Sweden to participate in a study examining SARS-Cov-2 antibodies in relation to registered sick leave. For consenting 12,928 healthy hospital employees antibodies to SARS-CoV-2 could be determined and compared to participant sick leave records. Subjects with viral serum antibodies were not at excess risk for future sick leave (adjusted odds ratio (OR) controlling for age and sex: 0.85 [95% confidence interval (CI) (0.85 (0.43–1.68)]. By contrast, subjects with antibodies had an excess risk for sick leave in the weeks prior to testing [adjusted OR in multivariate analysis: 3.34 (2.98–3.74)]. Thus, presence of viral antibodies marks past disease and protection against excess risk of future disease. Knowledge of whether exposed subjects have had disease in the past or are at risk for future disease is essential for planning of control measures.

Trial registration: First registered on 02/06/20, ClinicalTrials.gov NCT04411576.

## Introduction

To design strategies for SARS-CoV-2 control, knowledge of whether exposed individuals are immune against future disease is critical^1^. The incubation time from exposure to onset of symptoms has been estimated to last a median of six days, with peak infectiousness occurring zero to two days before onset of symptoms^2^ and pre-symptomatic spread is estimated to account for a substantial proportion of disease transmission^2,3^. Infectiousness decreases with increasing time after onset of symptoms^4^ and some individuals may remain asymptomatic despite being virus positive^5^. The IgG response develops rather slowly, commonly concomitantly with symptom resolution and then increases in subsequent weeks^6^. One report found that all COVID-19 patients had become seropositive 19 days after onset of symptoms^7^. Although there are many studies on viral antibodies and immunity, the extent and duration of immunity and the predictive value of presence of viral antibodies is still uncertain. In addition to the important issue of whether antibodies protect against a new infection, it is also important to know whether antibodies predict future disease or not. Future disease could conceivably also be caused by relapse of symptomatic disease or by late-onset symptomatic disease occurring after the antibodies have appeared. A problem is that studies that are based on past sickness are fraught with recall biases (subjects knowing their antibody status preferentially recalling events) and that prospective studies using future sickness as endpoint need to be very large.

Therefore, we wished to assess to what extent antibodies predicted future disease, using a large cohort of healthcare workers (HCWs) where data on past sickleave had already been collected and that could be followed up using the same administrative system in a manner free from recall bias. Immunity to other coronaviruses is known to be short-lasting^8^ and it has been argued that by analogy also SARS-CoV-2 immunity is likely to be shortlasting.

A secondary aim of the study—added after we realized that we had cohort follow-up data for almost 8 months and a second outbreak of SARS-CoV-2 had occurred—was therefore to investigate if the seropositivity measured during the first outbreak would affect the likelihood of disease also on the long term.

## Results

**Figure 1:**
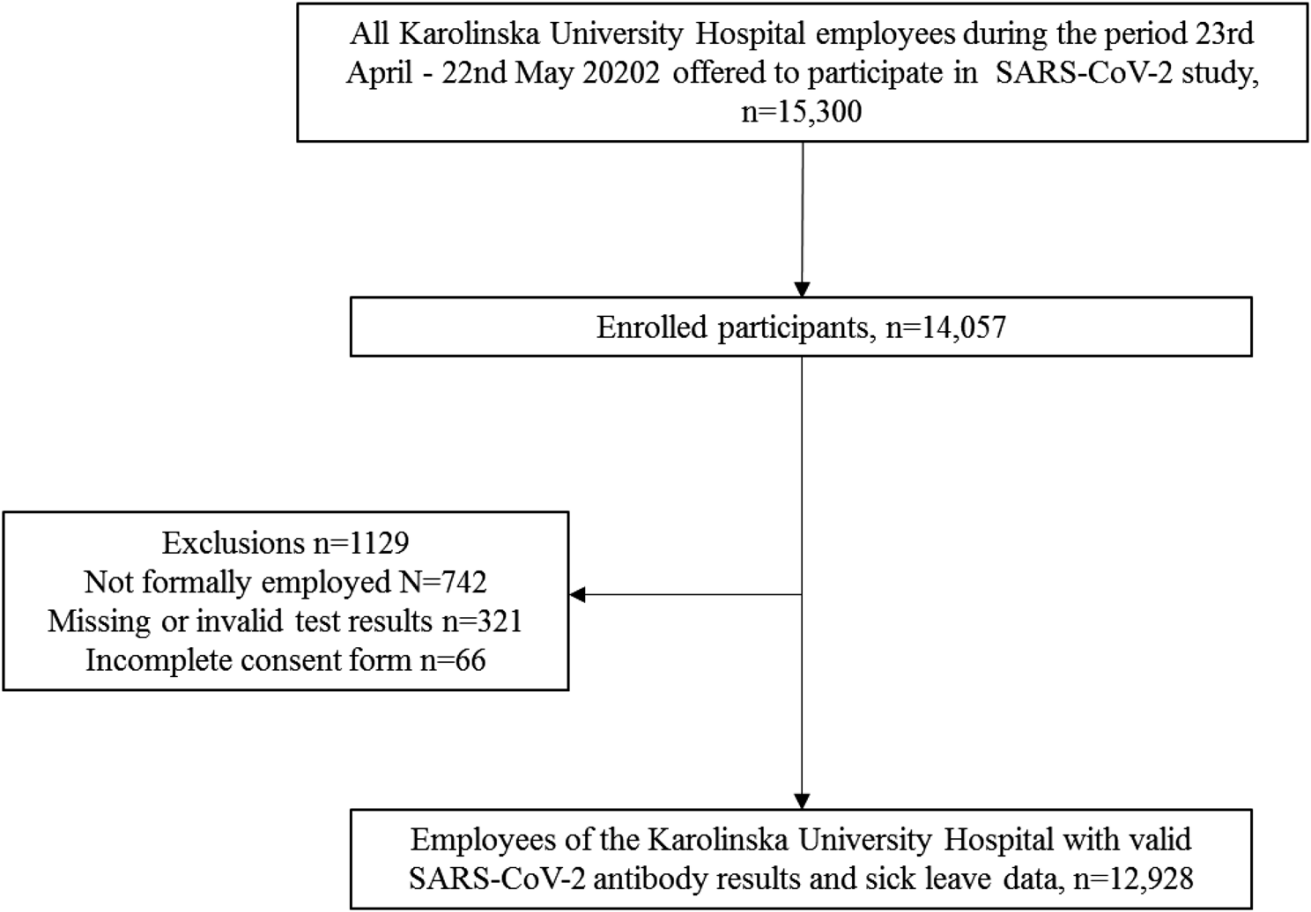
STROBE flowchart of study participants.

We invited all employees currently at work at the Karolinska University Hospital in Stockholm, Sweden (n = 15,300) to participate in a longitudinal cohort study of SARS-CoV-2 testing in relation to both past and future sick leave. We enrolled 14,052 participants (Fig. 1). After exclusion of participants not formally employed (e.g. medical students) and subjects with invalid tests, the final cohort consisted of 12,928 subjects with complete data on sick leave and valid SARS-CoV-2 antibody results (Fig. 1).

The overall number and proportion of employees that tested positive for antibodies to SARS-CoV-2 are shown by age in ten-year spans in Table 1. The proportion of serology-positive subjects was greatest among the youngest employees and decreased significantly with increasing age (*p* value for trend < 0.0001). The employees in the cohort that reported no patient contact whatsoever (n = 3285) were used as a reference cohort for estimation of the approximate general spread of the infection in the region. As detailed elsewhere^9^, the reference cohort had a seroprevalence of 9.9% that increased slightly during the time of the study, suggesting that most of the outbreak had already occurred in the weeks before the study started.

**Table 1:** Detection of antibodies to the SARS CoV-2 virus among 12,928 employees of the Karolinska University Hospital, by age.

Age	Serology positive		Total
	n	% (95% CI)^a^	
< 29	249	16.4 (14.6–18.3)	1522
30–39	383	12.1 (11.0–13.3)	3172
40–49	370	11.4 (10.4–12.6)	3238
50–59	313	10.2 (9.2–11.3)	3066
60+	166	8.6 (7.4–9.9)	1930
Total	1481	11.5 (10.9–11.8)	12,928

^a^Cochran–Armitage trend test for decreasing prevalence with increasing age, *p* value < 0.0001.

Positivity in serology was significantly associated with an excess risk for having been on sick leave in the past 6 weeks but did not confer any excess risk for future sick leave for the coming two weeks after testing (Table 2). The mutual adjustments (age, sex, and serostatus) in the multivariate model had only minor effects on the estimates (Supplementary Table 2).

**Table 2:** Association between covariates and sick leave, mutually adjusted.

	Up to 2 weeks after testing versus no sick leave OR (95% CI)	Up to 6 weeks before testing versus no sick leave OR (95% CI)
**Age**		
< 29	1.00	1.00
30–39	1.05 (0.64–1.74)	1.16 (1.02–1.32)
40–49	0.65 (0.38–1.11)	1.04 (0.91–1.18)
50–59	0.61 (0.35–1.06)	0.97 (0.85–1.10)
60+	0.47 (0.24–0.90)	0.82 (0.71–0.95)
**Sex**		
Female	1.00	1.00
Male	0.46 (0.28–0.74)	0.58 (0.53–0.64)
**SARS-CoV-2 antibody status**		
Negative	1.00	1.00
Positive	0.85 (0.43–1.68)	3.34 (2.98–3.74)

**Figure 2:**
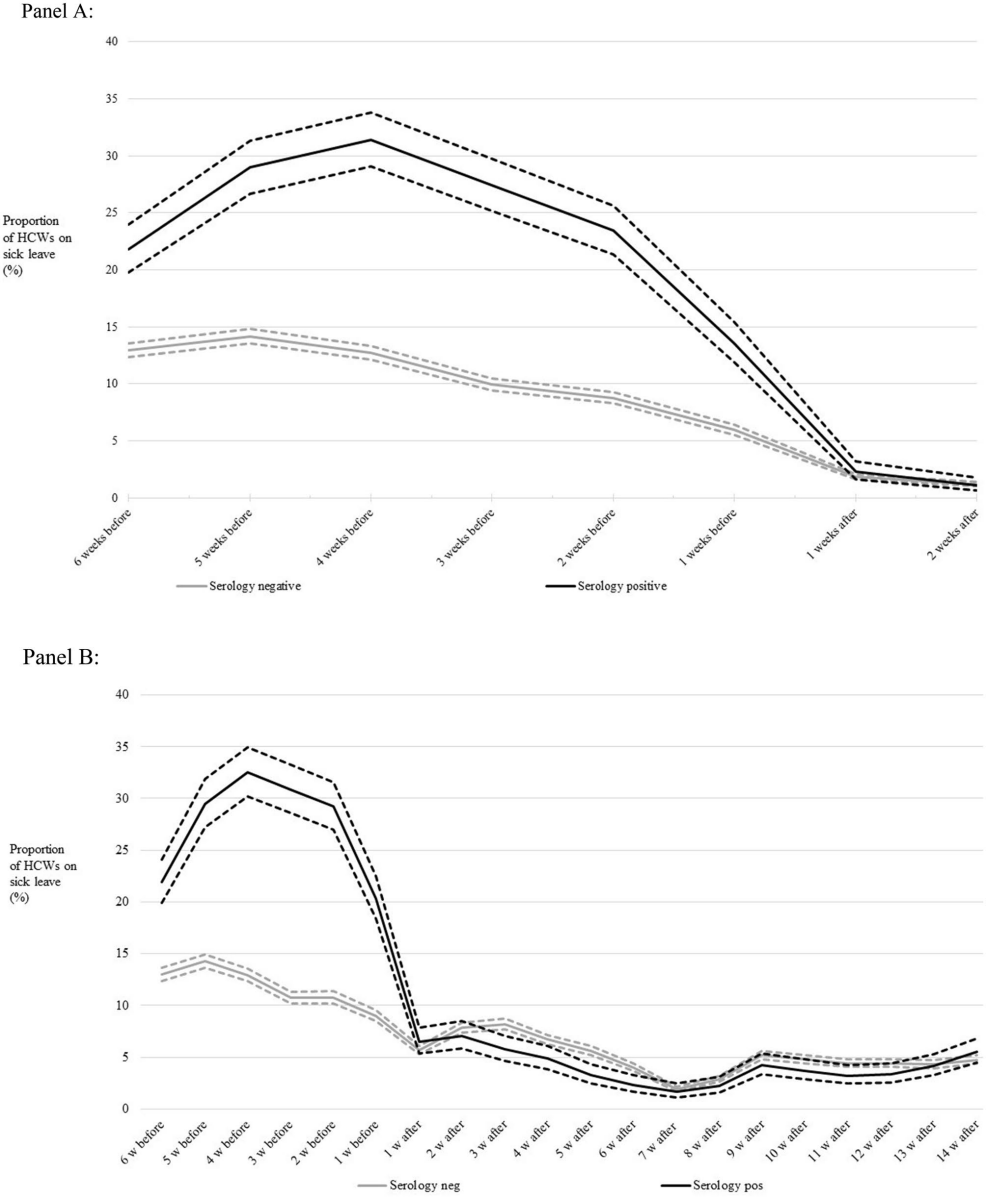
Proportion (%) of healthcare workers on sick leave during the study period, by serology test result. Dashed lines around the lines denote the 95% confidence intervals. (**A**) Follow-up restricted to the 2 weeks after testing when participants had no knowledge of testing results. (**B**) Follow-up up to 14 weeks after testing.

As Covid-19 is known to preferentially affect older infected subjects and have a long sickness duration, we next analyzed the length of sick leave in relation to age and serology status (Table 3). No past sick leave was found for 66% of the antibody-negative subjects, whereas only 35% of the antibody-positive subjects had no past sick leave (Table 3). The association of seropositivity with any sick leave in the past was highly significant [OR: 3.34 (2.98–3.74)] and mostly due to sick leave longer than five days (Table 3). Figure 2 displays the proportion of participants on sick leave in relation to their antibody status. The typical seasonal pattern with reported sick leave declining over time during the spring was seen for both seronegative and seropositive subjects (Fig. 2A). Comparison with sick leave data for 2019 found that the sick leave pattern by calendar week for seronegative subjects in 2020 was almost identical to the sick leave pattern by calendar week for all employees during 2019 (not shown). The proportion of seropositive health care workers on sick leave peaked at four weeks before testing and then decreased to the same level as the seronegative subjects in the weeks after testing. This is in line with our finding in the multinomial logistic regression analysis that there was no excess risk for future sick leave in the weeks following testing (Table 2). The elevated excess odds ratio for past sick leave was similar for the different weeks before testing (estimates not shown).

**Table 3:** Duration of sick leave in days, by SARS-CoV-2 serology test result and age.

Antibody status	Age	Cumulative number of sick leave days over the period^a^				
		0 days n (%)	1–5 days n (%)	6–10 days n (%)	11–15 days n (%)	≥ 16 days n (%)
Negative	< 29	857 (67.3)	312 (24.5)	78 (6.1)	13 (1.0)	13 (1.0)
	30–39	1815 (65.1)	677 (24.3)	209 (7.5)	57 (2.0)	31 (1.1)
	40–49	1934 (67.4)	617 (21.5)	212 (7.4)	54 (1.9)	51 (1.8)
	50–59	1897 (68.9)	519 (18.9)	211 (7.7)	77 (2.8)	49 (1.8)
	60+	1282 (72.7)	287 (16.3)	119 (6.8)	46 (2.6)	30 (1.7)
	Total n, % (95% CI)	7875, 68.8 (67.9–69.6)	2412, 21.1 (20.3–21.8)	829, 7.2 (6.8–7.7)	247, 2.2 (1.9–2.4)	174, 1.5 (1.3–1.7)
Positive	< 29	109 (43.8)	63 (25.3)	55 (22.1)	16 (6.4)	6 (2.4)
	30–39	143 (37.3)	117 (30.6)	79 (21.4)	36 (9.4)	8 (2.1)
	40–49	143 (38.7)	97 (26.2)	79 (21.4)	42 (11.4)	9 (2.4)
	50–59	113 (36.1)	60 (19.2)	89 (28.4)	29 (9.3)	22 (7.0)
	60+	68 (41.0)	33 (19.9)	37 (22.3)	21 (12.7)	7 (4.2)
	Total n, % (95% CI)	576, 38.9 (36.4–41.4)	370, 25.0 (22.9–27.3)	339, 22.9 (20.8–25.1)	144, 9.7 (8.3–11.3)	52, 3.5 (2.7–4.6)

^a^Sick leave days in the 6 weeks prior to testing.

To investigate if the low risk for future sick leave would persist also after the first 2 weeks of follow-up, the analysis was extended to 14 weeks after testing. There are several possible ways whereby participants’ knowledge of test results may create bias and the analysis in Fig. 2A with follow-up only when participants were unaware of testing results is therefore our main analysis. However, the analysis up to 14 weeks post testing found very similar results (Fig. 2B) suggesting that disclosure of testing results did not induce any major biases. The seropositive subjects have a significantly lower rate of sick leave up to 6 weeks post testing (non-overlapping confidence intervals in (Fig. 2B). From 7 to 14 weeks post testing the sick leave rates are very similar, but at that time there was very little SARS-CoV-2 infection in the region and comparison of sick leave rates if there is little SARS-CoV-2 infection was not meaningful.

In the last week of September 2020, a second outbreak of SARS-CoV-2 infection started. There were 2 major differences in the setting compared to the first outbreak. First, there was now widespread free of charge SARS-CoV-2 testing available. Second, a recommendation had been issued that household members of confirmed cases should self-isolate at home even if they were not sick. There was thus a surge in sick leave among subjects without symptoms. This had not been present during the first outbreak. We therefore analysed long-term immunity by first capturing all sick leave occurring during the last week of September up to 17th December 2020 and then analysed whether there had been a positive PCR test for SARS-CoV-2 within 7 days of the sick leave. There were 9882 participants that were still employed on 17th December 2020 (1136 had been seropositive and 8746 seronegative when tested during the first outbreak). There were 4 cases of disease among the seropositives and 77 cases among the seronegatives (OR: 0.40 (95% CI 0.123–0.996), *p* < 0.05. Conditional maximum likelihood estimate of Odds Ratio with Mid-P exact confidence limits).

## Discussion

We provide large-scale data that antibodies to the SARS-CoV-2 virus associated with protection against excess risk of future sick leave in a large, well-defined cohort of hospital employees. Antibodies were instead associated with past sick leave and serology was thus found to be useful for distinguishing whether subjects who are exposed to the virus may be at risk for development of virus-associated disease or not. Although we find that seropositive subjects had no excess risk of future disease, this is not the same thing as immunity. Disease could conceivably be induced by recurrent disease originating from the same infection or be the result of a late-onset symptomatic disease starting after the antibodies were induced. Our data does suggest that recurrent or late-onset disease among seropositives is not common.

Our long-term follow-up suggested some protection against SARS-CoV-2 verified disease occurring during the second outbreak 4–8 months later. However, there were only 81 cases in this analysis and biases induced by knowledge of the earlier serology results are possible. Furthermore, if there is some long-term immunity this does not imply that it is mediated by the antibodies as it could e.g. be associated with a resolved disease that has induced cellular immunity.

Strengths of our study include the fact that it was a large and systematically enrolled cohort that used administrative sick leave data and was therefore not hampered by the recall bias to which studies collecting self-reported information from participants can be subjected. We used a serology platform that contained several SARS-CoV-2 proteins and was validated to have very high sensitivity and specificity as a marker for PCR-verified SARS-CoV-2 infection. Test results were delivered to participants, but as the analyses took more than two weeks to complete, registered sick leave in the first weeks after enrollment occurred before the results were delivered. The strong protective effect of seropositivity against sick leave in the weeks after testing could thus not have been induced by knowledge of the test results.

A limitation is that we were not able to study infections occurring more than six to seven weeks before enrollment, as community transmission of SARS-CoV-2 in the region started only about six to seven weeks before the study. Participants were not surveyed about present or prior symptoms, but the hospital rules were clear that employees with symptoms should not be at work and we had, by design, decided to use only sick leave data to avoid possible recall bias. Hospital rules state that also employees working from home that develop COVID-19 symptoms must report this as sick leave. SARS-CoV-2 testing was not widely available at the time of the first outbreak and we therefore do not know which instances of sick leave were caused by SARS-CoV-2. However, the huge excess in sick leave during the outbreak, the typical characteristics (long duration especially among older subjects) and the strong association with seropositivity does indicate that most of the excess sick leave was SARS-CoV-2 related. Also, all comparisons are made against a baseline of sick leave among seronegatives that was very similar to the sick leave data from 2019.

We conclude that antibody testing is a powerful tool for identification of subjects who have had prior virus exposure but do not have an excess risk of disease. Testing for the viral nucleic acid by PCR will be positive also for asymptomatic subjects (including those in the pre-symptomatic phase who develop disease later), the date of exposure is for most subjects not known and the date of onset of symptoms can only be queried if there has been symptomatic disease.

We would like to caution that there is a large number of serology tests currently on the market and the extent of their validation may vary. Also, none of these tests have been specifically validated for the indication proposed here, namely, to separate exposed subjects who do not have excess risk of future disease from exposed subjects at risk to develop disease in the future.

In summary, our main finding suggests that validated antibody testing may be helpful in SARS-CoV-2 screening strategies as antibody-positive subjects were found to have no excess risk for future disease.

## Methods

There was an outbreak of SARS-CoV-2 in Stockholm, Sweden in March–May 2020. The outbreak was recognized on 13th of March, but as the first fatality occurred on 17th of March the outbreak had probably started somewhat earlier. During the time of this study, there was only limited SARS-CoV-2 testing but analysis of excess mortality data indicate that most of the outbreak was over by the second week of May and that there was no longer any excess mortality after the last week of June^10^.

The Karolinska University Hospital is one of the largest university hospitals in Europe, with about 15,300 employees (employment during enrollment, 23rd April–22nd May 2020). The hospital announced that all employees who were at work (and not on sick leave) were welcome to participate in a study about SARS-CoV-2. We enrolled 14,057 participants who all signed a written informed consent that also included permission to extract data from the employer’s administrative databases that contain data on sick leave. Most participants were between 30 and 59 years old and 79% were females (Supplementary Table 1). Employees were not allowed to work in case of symptoms and should have been free of symptoms for 48 h before returning to work. Analysis results were efficiently reported to participants by SMS, but analyses took 4 weeks post sampling to complete. Sick leave data was obtained from 6 weeks before sampling until 2 weeks after sampling using the personal identity numbers of the study subjects linked individually to the administrative systems of the hospital. A subcohort of 3285 of the enrolled employees reported having had no contact with patients whatsoever and were used as a reference cohort^9^.

In the last week of September 2020, a second outbreak of SARS-CoV-2 became evident in the region. The second outbreak is still ongoing at the time of writing.

### Ethics declaration

The study was approved by the National Ethical Review Agency of Sweden (Decision number 2020-01620). Trial registration number: ClinicalTrials.gov NCT04411576. All methods were carried out in compliance with the Helsinki declaration.

### Analyses of SARS-CoV-2 antibodies

Whole blood was collected in serum-separating tubes and centrifuged under 2000×*g* for ten minutes. Serum samples were inactivated by heating to 56 °C for 30 min and then stored at minus 20 °C until further analysis.

Different SARS-CoV-2 protein constructs and different production hosts were compared for expression of viral proteins using the mammalian HEK cell line as starting point. The evaluation of different production hosts was based on degree of concordance in antibody reactivity of the alternative hosts with the virus proteins produced in the HEK cells. Thereafter, the most efficient production and purification pipeline was chosen. Consequently, antigen reactivity was measured towards three different virus protein variants, (1) Spike trimers comprising the prefusion-stabilized spike glycoprotein ectodomain^11^ expressed in HEK cells and purified using a C-terminal Strep II tag), (2) Spike S1 domain, expressed in CHO cells and purified using C-terminal HPC4-tag, and (3) Nucleocapsid protein, expressed in *E.coli* and purified using a C-terminal His-tag.

The sera were analyzed using a multiplex antigen bead array in high throughput 384-plates format using a FlexMap3D instrument (Luminex Corp) with IgG detection^12^. The cut-off for seropositivity was for each antigen defined as mean + 6SD of 12 negative control samples included in each analysis batch. To be assigned as IgG positive, a sample was required to show reactivity against at least two of the three included viral antigens. Serum IgG bound to antigen coated beads was detected by F(ab′)2-Goat anti-Human IgG Fc Secondary Antibody, PEfluorescent anti-hIgG (Invitrogen, H10104. Validation procedure is described at www.thermofisher.com/se/en/home/life-science/antibodies/invitrogen-antibody-validation.html) and recorded as relative fluorescence intensity (AU). Four positive controls were re-run on every assay-plate and had a mean inter-assay coefficient of variation of 10.1% (8.0–13.3%), based on absolute intensity levels.

The serology assay was evaluated based on the analyses of 243 samples from Covid-19 subjects (defined as PCR-positive individuals sampled more than 16 days after positive PCR test) and 442 negative control samples (defined as samples collected 2019 or earlier, including 26 individuals with confirmed infections of other Coronaviruses than SARS-CoV-2). Based on these samples, the assay had a 99.2% sensitivity and 99.8% specificity.

### Data analyses

With conventional statistical power and two-sided tests of significance, and assuming a cumulative proportion of sick leave among non-exposed persons of 30% and that 10% of the cohort might be exposed, at least 3800 subjects would need to be enrolled to be able to detect associations of 1.4 or greater, a level which was considered to be medically meaningful.

Descriptive statistics examined test results by age, sex, and sick leave. A Cochran-Armitage Trend Test was used to examine patterns of seropositivity by age. Cumulative sick leave was examined in the six weeks prior to testing as the total number of days on sick leave for each subject, categorized as 0 days (no sick leave prior to testing), 1–5 days, 6–10 days, 11–15 days, and more than 16 days. Cumulative sick leave in days was then shown by age group stratified for seropositivity and negativity. A multinomial logistic regression model was used to examine the association between serology test results and sick leave measured as a categorical variable with three levels, no sick leave (reference category), sick leave in the 2 weeks after testing, and sick leave in the 6 weeks prior to testing. For subjects with sick leave in more than one category, the period with the most days on sick leave was chosen. If two periods had an equal number of sick leave days, the period further back in time was chosen. The final model was adjusted for age in ten-year categories and gender. Analyses were completed using SAS 9.4, Cary, NC.

## Supplementary Information

Supplementary Tables.

## Data Availability

The data constitutes sensitive data about health of human research subjects and thus cannot be directly deposited openly. However, pseudonymised, individual-level data that allow full replication of the results in this article are freely available from joakim.dillner@sll.se or from the Karolinska University Hospital data analysis department: tableau.karolinska@sll.se.
