# Supplementary material for: Antibodies to SARS-CoV-2 and risk of past or future sick leave
Source: Sci Rep. 2021 Mar 4;11:5160. doi: 10.1038/s41598-021-84356-w (PMC7933367; doi:10.1038/s41598-021-84356-w)
Supplement: Supplementary file 1 — Supplementary Tables. [file 41598_2021_84356_MOESM1_ESM.docx]

**Title: Antibodies to SARS-CoV-2 and risk of future sickness**

**Authors:**  Joakim Dillner^1*^, K. Miriam Elfström^1^, Jonas Blomqvist^6^, Carina Eklund^1^, Camilla Lagheden^1^, Sara Nordqvist-Kleppe^1^, Cecilia Hellström^4^, Jennie Olofsson^4^, Eni Andersson^4^, August Jernbom Falk^4^, Sofia Bergström^4^, Emilie Hultin^1^, Elisa Pin^4^, Anna Månberg^4^, Peter Nilsson^4^, My Hedhammar^5^, Sophia Hober^5^, Johan Mattsson^6^, Laila Sara Arroyo Mühr^1^,

Kalle Conneryd Lundgren^6^

**Table S1. Distribution of covariates**

|  | **n, % (95% CI)** | **Sick leave** | | |
| --- | --- | --- | --- | --- |
|  |  | **No sick leave  n (%)** | **0-2 weeks after testing n (%)** | **0-6 weeks before testing n (%)** |
| **Age** | | | | |
| <29 | 1,522 (11.8) | 945 (62.1) | 23 (1.5) | 554 (36.4) |
| 30-39 | 3,172 (24.6) | 1,918 (60.5) | 48 (1.5) | 1,206 (38.0) |
| 40-49 | 3,238 (25.1) | 2,049 (63.3) | 32 (1.0) | 1,157 (35.7) |
| 50-59 | 3,066 (23.7) | 1,985 (64.7) | 30 (1.0) | 1,051 (34.3) |
| 60+ | 1930 (14.9) | 1,337 (69.3) | 15 (0.8) | 578 (30.0) |
| **Sex** | | | | |
| Female | 10,203 (78.9) | 6,248 (61.2) | 129 (1.3) | 3,826 (37.5) |
| Male | 2,725 (21.1) | 1,986 (72.9) | 19 (0.7) | 720 (26.4) |
| **SARS-CoV-2 Antibody status** | | | | |
| Negative | 11,447 (88.5) | 7,664 (67.0) | 139 (1.2) | 3,644 (31.8) |
| Positive | 1,481 (11.5) | 570 (38.5) | 9 (0.6) | 902 (60.9) |

**Table S2. Bivariate association between covariates and sick leave (not mutually adjusted)**

|  | **0-2 weeks after testing vs No sickleave OR (95% CI)** | **0-6 weeks before testing vs No sickleave OR (95% CI)** |
| --- | --- | --- |
| **Age** | | |
| <29 | 1,00 | 1,00 |
| 30-39 | 1.03 (0.62-1.70) | 1.07 (0.94-1.22) |
| 40-49 | 0.64 (0.37-1.10) | 0.96 (0.85-1.09) |
| 50-59 | 0.62 (0.36-1.07) | 0.90 (0.79-1.03) |
| 60+ | 0.46 (0.24-0.89) | 0.74 (0.64-0.85) |
| **Sex** | | |
| Female | 1,00 | 1,00 |
| Male | 0.46 (0.29-0.75) | 0.59 (0.54-0.65) |
| **SARS-CoV-2 Antibody Status** | | |
| Negative | 1,00 | 1,00 |
| Positive | 0.87 (0.44-1.72) | 3.33 (2.98-3.72) |
